# Poly[triaqua­[μ_4_-3-(4-carboxyl­atophen­oxy)propionato-κ^4^ *O*:*O*′:*O*′′:*O*′′′][μ_3_-3-(4-carboxyl­atophen­oxy)propionato-κ^3^ *O*:*O*′:*O*′′]dizinc]

**DOI:** 10.1107/S1600536812003200

**Published:** 2012-01-31

**Authors:** Shan Gao, Seik Weng Ng

**Affiliations:** a Key Laboratory of Functional Inorganic Material Chemistry, Ministry of Education, Heilongjiang University, Harbin 150080, People’s Republic of China; b Department of Chemistry, University of Malaya, 50603 Kuala Lumpur, Malaysia; c Chemistry Department, Faculty of Science, King Abdulaziz University, PO Box 80203 Jeddah, Saudi Arabia

## Abstract

The coordination polymer [Zn_2_(C_10_H_8_O_5_)_2_(H_2_O)_3_]_*n*_ adopts a layer structure in which the two independent Zn^II^ ions exist in trigonal–bipyramidal coordination geometries. One carboxyl­ate dianion binds to a monoaqua-coordinated metal ion through the aliphatic carboxyl­ate end and to the diaqua-coordinated metal ion through the aromatic carboxyl­ate end; the other dianion binds in the reverse manner. Three of the four carboxyl­ate ends of the two dianions are also engaged in bridging inter­actions; these lead to a layer structure parallel to (100). Adjacent layers are linked by O—H_water_⋯O hydrogen bonds into a three-dimensional network.

## Related literature

For the cobalt(II) derivative of 3-(4-carb­oxy­phen­oxy)­propionic acid, see: Xiao *et al.* (2006 ▶).
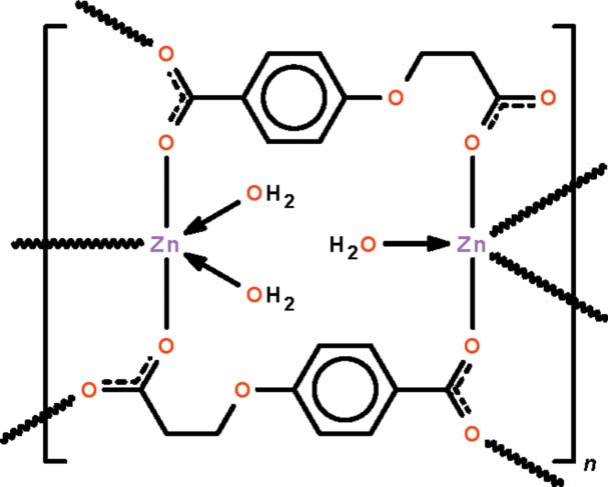


## Experimental

### 

#### Crystal data

[Zn_2_(C_10_H_8_O_5_)_2_(H_2_O)_3_]
*M* *_r_* = 601.12
Triclinic, 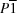

*a* = 7.6518 (3) Å
*b* = 11.3553 (5) Å
*c* = 13.5294 (6) Å
α = 77.2436 (14)°
β = 85.6236 (13)°
γ = 73.3105 (13)°
*V* = 1098.13 (8) Å^3^
*Z* = 2
Mo *K*α radiation
μ = 2.26 mm^−1^
*T* = 293 K
0.17 × 0.13 × 0.11 mm

#### Data collection

Rigaku R-AXIS RAPID IP diffractometer
Absorption correction: multi-scan (*ABSCOR*; Higashi, 1995 ▶) *T*_min_ = 0.700, *T*_max_ = 0.790
10809 measured reflections
4954 independent reflections
4160 reflections with *I* > 2σ(*I*)
*R*_int_ = 0.019

#### Refinement

*R*[*F* ^2^ > 2σ(*F* ^2^)] = 0.026
*wR*(*F* ^2^) = 0.074
*S* = 1.04
4954 reflections
334 parameters
9 restraints
H atoms treated by a mixture of independent and constrained refinement
Δρ_max_ = 0.45 e Å^−3^
Δρ_min_ = −0.45 e Å^−3^

### 

Data collection: *RAPID-AUTO* (Rigaku, 1998 ▶); cell refinement: *RAPID-AUTO*; data reduction: *CrystalClear* (Rigaku, 2002 ▶); program(s) used to solve structure: *SHELXS97* (Sheldrick, 2008 ▶); program(s) used to refine structure: *SHELXL97* (Sheldrick, 2008 ▶); molecular graphics: *X-SEED* (Barbour, 2001 ▶); software used to prepare material for publication: *publCIF* (Westrip, 2010 ▶).

## Supplementary Material

Crystal structure: contains datablock(s) global, I. DOI: 10.1107/S1600536812003200/bt5797sup1.cif

Structure factors: contains datablock(s) I. DOI: 10.1107/S1600536812003200/bt5797Isup2.hkl

Additional supplementary materials:  crystallographic information; 3D view; checkCIF report

## Figures and Tables

**Table 1:** Hydrogen-bond geometry (Å, °)

*D*—H⋯*A*	*D*—H	H⋯*A*	*D*⋯*A*	*D*—H⋯*A*
O1*W*—H11⋯O4^i^	0.83 (1)	1.99 (1)	2.781 (2)	159 (2)
O1*W*—H12⋯O4^ii^	0.83 (1)	1.94 (1)	2.768 (2)	173 (2)
O2*W*—H21⋯O4^iii^	0.83 (1)	2.13 (2)	2.854 (2)	145 (2)
O2*W*—H22⋯O7^iv^	0.84 (1)	1.98 (1)	2.776 (2)	158 (2)
O3*W*—H31⋯O2^v^	0.83 (1)	1.91 (2)	2.700 (2)	158 (3)
O3*W*—H32⋯O9^vi^	0.84 (1)	2.31 (2)	3.026 (2)	143 (3)

Symmetry codes: (i) 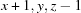; (ii) 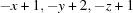; (iii) 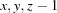; (iv) 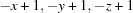; (v) 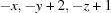; (vi) 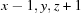.

**Fig. 1.**
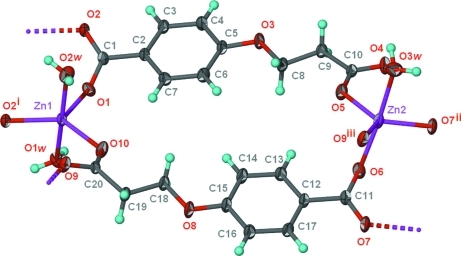
Anisotropic displacement ellipsoid plot (Barbour, 2001) of a portion of polymeric [Zn2(H2O)3(C10H8O5)2]n at the 50% probability level; hydrogen atoms are drawn as spheres of arbitrary radius.

## supplementary crystallographic information

### Comment

We reported the crystal structure of 3-(4-carboxyphenoxy)propropionic acid. We also reported the crystal structures of some metal derivatives. In the water-coordinated cobalt(II) derivative, two tetraaquacobalt cations are bridged by two 3-(4-carboxylatophenoxy)propionate dianions across a center of inversion to form a dinuclear molecule (Xiao *et al.*, 2006). The title coordination polymer (Scheme I) adopts a layer structure in which the two independent Zn^II^ atoms exist in trigonal bipyramidal geometries. One carboxylate dianion binds to a mono-aqua coordinated metal atom through the aliphatic carboxyl end and to the di-aqua coordinated metal atom through the aromatic carboxyl end; the other dianion binds in the reverse manner (Fig.1). Three of the four carboxyl ends of the two dianions are also engaged in bridging interactions; these lead to a layer structure. Adjacent layers are linked by O–H_water_···O hydrogen bonds into a three-dimensional network (Table 1).

### Experimental

Zinc acetate (1 mmol) and 3-(4-carboxyphenoxy)propionic acid (1 mmol) were dissolved in water (10 ml). Sodium hydroxide (1 *M*) was added in drops until the solution registered a pH of 7. The solution was then set aside for the growth of colorless crystals.

### Refinement

Carbon-bound H-atoms were placed in calculated positions (C–H 0.93–0.97 Å) and were included in the refinement in the riding model approximation with *U*(H) set to 1.2–1.5*U*(C). The water H-atoms were located in a difference Fouier map, and were refined with distance restraints of O–H 0.84±0.01 and H···H 1.37±0.01 Å; their displacement factors were set to 1.5U_eq_(O).

### Crystal data

**Table:**

[Zn_2_(C_10_H_8_O_5_)_2_(H_2_O)_3_]	*Z* = 2
*M**_r_* = 601.12	*F*(000) = 612
Triclinic, *P*1	*D*_x_ = 1.818 Mg m^−^^3^
Hall symbol: -P 1	Mo *K*α radiation, λ = 0.71073 Å
*a* = 7.6518 (3) Å	Cell parameters from 8993 reflections
*b* = 11.3553 (5) Å	θ = 3.1–27.5°
*c* = 13.5294 (6) Å	µ = 2.26 mm^−^^1^
α = 77.2436 (14)°	*T* = 293 K
β = 85.6236 (13)°	Prism, colorless
γ = 73.3105 (13)°	0.17 × 0.13 × 0.11 mm
*V* = 1098.13 (8) Å^3^	

### Data collection

**Table:**

Rigaku R-AXIS RAPID IP diffractometer	4954 independent reflections
Radiation source: fine-focus sealed tube	4160 reflections with *I* > 2σ(*I*)
graphite	*R*_int_ = 0.019
ω scan	θ_max_ = 27.5°, θ_min_ = 3.1°
Absorption correction: multi-scan (*ABSCOR*; Higashi, 1995)	*h* = −9→9
*T*_min_ = 0.700, *T*_max_ = 0.790	*k* = −14→14
10809 measured reflections	*l* = −17→17

### Refinement

**Table:**

Refinement on *F*^2^	Primary atom site location: structure-invariant direct methods
Least-squares matrix: full	Secondary atom site location: difference Fourier map
*R*[*F*^2^ > 2σ(*F*^2^)] = 0.026	Hydrogen site location: inferred from neighbouring sites
*wR*(*F*^2^) = 0.074	H atoms treated by a mixture of independent and constrained refinement
*S* = 1.04	*w* = 1/[σ^2^(*F*_o_^2^) + (0.0397*P*)^2^ + 0.4093*P*] where *P* = (*F*_o_^2^ + 2*F*_c_^2^)/3
4954 reflections	(Δ/σ)_max_ = 0.001
334 parameters	Δρ_max_ = 0.45 e Å^−^^3^
9 restraints	Δρ_min_ = −0.45 e Å^−^^3^

### Fractional atomic coordinates and isotropic or equivalent isotropic displacement parameters (Å^2^)

**Table:**

	*x*	*y*	*z*	*U*_iso_*/*U*_eq_	
Zn1	0.63992 (3)	0.83763 (2)	0.070119 (16)	0.02491 (8)	
Zn2	0.26901 (4)	0.60896 (2)	0.918931 (17)	0.03034 (8)	
O1	0.54606 (19)	0.99522 (13)	0.12262 (11)	0.0303 (3)	
O2	0.3096 (2)	1.16123 (14)	0.07830 (10)	0.0327 (3)	
O3	0.1731 (2)	1.04925 (14)	0.55209 (10)	0.0362 (4)	
O4	0.1231 (2)	0.89307 (14)	0.90191 (11)	0.0338 (3)	
O5	0.2209 (2)	0.75470 (14)	0.80329 (11)	0.0367 (4)	
O6	0.5517 (2)	0.57863 (14)	0.89038 (11)	0.0354 (4)	
O7	0.7337 (2)	0.38375 (14)	0.93679 (10)	0.0340 (3)	
O8	0.8141 (2)	0.43774 (14)	0.46231 (10)	0.0385 (4)	
O9	0.7217 (2)	0.55924 (13)	0.10884 (11)	0.0329 (3)	
O10	0.7013 (2)	0.70540 (13)	0.19529 (11)	0.0324 (3)	
O1W	0.9003 (2)	0.86744 (15)	0.07296 (12)	0.0335 (3)	
H11	0.959 (3)	0.860 (2)	0.0192 (13)	0.050*	
H12	0.888 (4)	0.9382 (14)	0.0853 (18)	0.050*	
O2W	0.3716 (2)	0.83032 (16)	0.06475 (12)	0.0380 (4)	
H21	0.328 (4)	0.870 (2)	0.0085 (12)	0.057*	
H22	0.361 (4)	0.7572 (12)	0.077 (2)	0.057*	
O3W	−0.0265 (2)	0.62923 (17)	0.93647 (14)	0.0452 (4)	
H31	−0.105 (3)	0.6968 (18)	0.9157 (19)	0.068*	
H32	−0.048 (4)	0.600 (3)	0.9969 (10)	0.068*	
C1	0.4045 (3)	1.07348 (18)	0.14434 (14)	0.0234 (4)	
C2	0.3465 (3)	1.06585 (18)	0.25246 (14)	0.0245 (4)	
C3	0.2374 (3)	1.17051 (19)	0.28675 (15)	0.0279 (4)	
H3	0.1999	1.2472	0.2413	0.033*	
C4	0.1841 (3)	1.16205 (19)	0.38719 (15)	0.0298 (4)	
H4	0.1133	1.2332	0.4092	0.036*	
C5	0.2360 (3)	1.0472 (2)	0.45583 (15)	0.0281 (4)	
C6	0.3438 (3)	0.9416 (2)	0.42285 (16)	0.0341 (5)	
H6	0.3781	0.8646	0.4681	0.041*	
C7	0.4005 (3)	0.9514 (2)	0.32176 (16)	0.0312 (5)	
H7	0.4751	0.8810	0.3002	0.037*	
C8	0.2217 (3)	0.93520 (19)	0.62779 (15)	0.0296 (4)	
H8A	0.3531	0.9033	0.6343	0.036*	
H8B	0.1750	0.8713	0.6108	0.036*	
C9	0.1340 (3)	0.97182 (19)	0.72457 (15)	0.0286 (4)	
H9A	0.1824	1.0365	0.7386	0.034*	
H9B	0.0040	1.0082	0.7139	0.034*	
C10	0.1618 (3)	0.86549 (19)	0.81669 (14)	0.0246 (4)	
C11	0.6559 (3)	0.4798 (2)	0.86980 (15)	0.0275 (4)	
C12	0.6964 (3)	0.46932 (19)	0.76157 (14)	0.0267 (4)	
C13	0.6249 (3)	0.57192 (19)	0.68433 (15)	0.0288 (4)	
H13	0.5507	0.6458	0.7007	0.035*	
C14	0.6621 (3)	0.5661 (2)	0.58321 (15)	0.0325 (5)	
H14	0.6153	0.6361	0.5324	0.039*	
C15	0.7701 (3)	0.4548 (2)	0.55855 (15)	0.0288 (4)	
C16	0.8419 (3)	0.3509 (2)	0.63521 (16)	0.0335 (5)	
H16	0.9131	0.2762	0.6187	0.040*	
C17	0.8074 (3)	0.3587 (2)	0.73571 (15)	0.0318 (5)	
H17	0.8584	0.2899	0.7866	0.038*	
C18	0.7454 (3)	0.54209 (19)	0.38040 (14)	0.0294 (4)	
H18A	0.7940	0.6114	0.3831	0.035*	
H18B	0.6133	0.5708	0.3838	0.035*	
C19	0.8078 (3)	0.49400 (19)	0.28414 (14)	0.0278 (4)	
H19A	0.9401	0.4677	0.2815	0.033*	
H19B	0.7653	0.4210	0.2854	0.033*	
C20	0.7379 (3)	0.59226 (18)	0.18999 (14)	0.0226 (4)	

### Atomic displacement parameters (Å^2^)

**Table:**

	*U*^11^	*U*^22^	*U*^33^	*U*^12^	*U*^13^	*U*^23^
Zn1	0.03290 (14)	0.02110 (12)	0.01676 (12)	−0.00311 (9)	0.00282 (9)	−0.00263 (9)
Zn2	0.04902 (16)	0.02167 (13)	0.01550 (12)	−0.00240 (10)	−0.00106 (10)	−0.00351 (9)
O1	0.0326 (8)	0.0254 (7)	0.0311 (8)	−0.0037 (6)	0.0074 (6)	−0.0105 (6)
O2	0.0363 (8)	0.0361 (8)	0.0169 (7)	0.0019 (6)	0.0014 (6)	−0.0039 (6)
O3	0.0516 (10)	0.0296 (8)	0.0173 (7)	−0.0020 (7)	0.0065 (6)	0.0012 (6)
O4	0.0487 (9)	0.0341 (8)	0.0196 (7)	−0.0153 (7)	0.0066 (6)	−0.0047 (6)
O5	0.0563 (10)	0.0235 (7)	0.0211 (7)	−0.0011 (7)	0.0014 (7)	0.0002 (6)
O6	0.0450 (9)	0.0317 (8)	0.0274 (8)	−0.0068 (7)	0.0057 (7)	−0.0088 (6)
O7	0.0493 (9)	0.0333 (8)	0.0146 (7)	−0.0046 (7)	0.0004 (6)	−0.0044 (6)
O8	0.0595 (10)	0.0306 (8)	0.0141 (7)	0.0012 (7)	0.0011 (7)	0.0001 (6)
O9	0.0518 (9)	0.0240 (7)	0.0197 (7)	−0.0048 (7)	−0.0053 (6)	−0.0037 (6)
O10	0.0538 (9)	0.0205 (7)	0.0219 (7)	−0.0101 (6)	−0.0048 (7)	−0.0007 (6)
O1W	0.0325 (8)	0.0375 (9)	0.0313 (8)	−0.0102 (7)	0.0100 (6)	−0.0117 (7)
O2W	0.0416 (9)	0.0407 (9)	0.0327 (9)	−0.0189 (8)	−0.0044 (7)	0.0014 (7)
O3W	0.0379 (9)	0.0383 (10)	0.0460 (11)	−0.0004 (7)	0.0048 (8)	0.0038 (8)
C1	0.0284 (10)	0.0228 (9)	0.0205 (9)	−0.0086 (8)	0.0031 (8)	−0.0068 (7)
C2	0.0279 (10)	0.0259 (10)	0.0179 (9)	−0.0056 (8)	0.0015 (7)	−0.0036 (7)
C3	0.0372 (11)	0.0221 (9)	0.0198 (9)	−0.0042 (8)	0.0003 (8)	−0.0004 (7)
C4	0.0398 (11)	0.0230 (10)	0.0214 (10)	−0.0017 (8)	0.0026 (8)	−0.0039 (8)
C5	0.0333 (11)	0.0305 (11)	0.0172 (9)	−0.0071 (8)	0.0017 (8)	−0.0013 (8)
C6	0.0440 (13)	0.0238 (10)	0.0244 (10)	−0.0011 (9)	0.0019 (9)	0.0041 (8)
C7	0.0356 (11)	0.0246 (10)	0.0261 (10)	0.0003 (8)	0.0042 (9)	−0.0031 (8)
C8	0.0382 (11)	0.0270 (10)	0.0181 (9)	−0.0056 (9)	0.0010 (8)	0.0016 (8)
C9	0.0370 (11)	0.0249 (10)	0.0197 (9)	−0.0068 (8)	0.0014 (8)	0.0012 (8)
C10	0.0265 (10)	0.0272 (10)	0.0187 (9)	−0.0083 (8)	0.0011 (7)	−0.0009 (8)
C11	0.0335 (10)	0.0298 (10)	0.0196 (9)	−0.0101 (8)	0.0021 (8)	−0.0051 (8)
C12	0.0338 (11)	0.0292 (10)	0.0164 (9)	−0.0092 (8)	0.0012 (8)	−0.0033 (8)
C13	0.0363 (11)	0.0243 (10)	0.0231 (10)	−0.0049 (8)	0.0004 (8)	−0.0042 (8)
C14	0.0469 (13)	0.0271 (10)	0.0183 (9)	−0.0057 (9)	−0.0013 (9)	0.0003 (8)
C15	0.0392 (11)	0.0289 (10)	0.0165 (9)	−0.0079 (9)	0.0005 (8)	−0.0028 (8)
C16	0.0436 (12)	0.0276 (11)	0.0218 (10)	−0.0006 (9)	0.0022 (9)	−0.0027 (8)
C17	0.0392 (12)	0.0283 (11)	0.0204 (10)	−0.0017 (9)	−0.0007 (9)	0.0005 (8)
C18	0.0416 (12)	0.0264 (10)	0.0158 (9)	−0.0068 (9)	−0.0003 (8)	0.0005 (8)
C19	0.0395 (11)	0.0228 (10)	0.0166 (9)	−0.0051 (8)	−0.0003 (8)	0.0006 (7)
C20	0.0267 (9)	0.0236 (9)	0.0155 (8)	−0.0071 (7)	0.0032 (7)	−0.0010 (7)

### Geometric parameters (Å, °)

**Table:**

Zn1—O1	1.9919 (14)	C2—C7	1.397 (3)
Zn1—O2^i^	2.0136 (14)	C3—C4	1.379 (3)
Zn1—O10	1.9840 (14)	C3—H3	0.9300
Zn1—O1W	2.1188 (15)	C4—C5	1.395 (3)
Zn1—O2W	2.0855 (16)	C4—H4	0.9300
Zn2—O5	1.9791 (14)	C5—C6	1.389 (3)
Zn2—O6	2.1093 (16)	C6—C7	1.393 (3)
Zn2—O9^ii^	2.0073 (15)	C6—H6	0.9300
Zn2—O7^iii^	1.9702 (14)	C7—H7	0.9300
Zn2—O3W	2.2055 (17)	C8—C9	1.511 (3)
O1—C1	1.250 (2)	C8—H8A	0.9700
O2—C1	1.268 (2)	C8—H8B	0.9700
O2—Zn1^i^	2.0136 (14)	C9—C10	1.512 (3)
O3—C5	1.356 (2)	C9—H9A	0.9700
O3—C8	1.435 (2)	C9—H9B	0.9700
O4—C10	1.253 (2)	C11—C12	1.497 (3)
O5—C10	1.258 (2)	C12—C13	1.390 (3)
O6—C11	1.250 (3)	C12—C17	1.399 (3)
O7—C11	1.280 (2)	C13—C14	1.388 (3)
O7—Zn2^iii^	1.9702 (14)	C13—H13	0.9300
O8—C15	1.361 (2)	C14—C15	1.392 (3)
O8—C18	1.435 (2)	C14—H14	0.9300
O9—C20	1.261 (2)	C15—C16	1.394 (3)
O9—Zn2^ii^	2.0073 (15)	C16—C17	1.383 (3)
O10—C20	1.251 (2)	C16—H16	0.9300
O1W—H11	0.833 (9)	C17—H17	0.9300
O1W—H12	0.834 (9)	C18—C19	1.514 (3)
O2W—H21	0.832 (9)	C18—H18A	0.9700
O2W—H22	0.836 (10)	C18—H18B	0.9700
O3W—H31	0.833 (10)	C19—C20	1.512 (2)
O3W—H32	0.835 (10)	C19—H19A	0.9700
C1—C2	1.487 (3)	C19—H19B	0.9700
C2—C3	1.393 (3)		
			
O10—Zn1—O1	103.22 (6)	C7—C6—H6	120.1
O10—Zn1—O2^i^	133.60 (6)	C6—C7—C2	120.67 (19)
O1—Zn1—O2^i^	122.33 (6)	C6—C7—H7	119.7
O10—Zn1—O2W	95.04 (7)	C2—C7—H7	119.7
O1—Zn1—O2W	88.80 (7)	O3—C8—C9	104.73 (16)
O2^i^—Zn1—O2W	94.31 (7)	O3—C8—H8A	110.8
O10—Zn1—O1W	89.29 (6)	C9—C8—H8A	110.8
O1—Zn1—O1W	85.51 (6)	O3—C8—H8B	110.8
O2^i^—Zn1—O1W	86.15 (6)	C9—C8—H8B	110.8
O2W—Zn1—O1W	173.51 (6)	H8A—C8—H8B	108.9
O7^iii^—Zn2—O5	126.07 (6)	C10—C9—C8	115.08 (17)
O7^iii^—Zn2—O9^ii^	115.44 (6)	C10—C9—H9A	108.5
O5—Zn2—O9^ii^	117.57 (6)	C8—C9—H9A	108.5
O7^iii^—Zn2—O6	98.39 (6)	C10—C9—H9B	108.5
O5—Zn2—O6	89.55 (7)	C8—C9—H9B	108.5
O9^ii^—Zn2—O6	91.48 (6)	H9A—C9—H9B	107.5
O7^iii^—Zn2—O3W	86.84 (7)	O4—C10—O5	123.89 (18)
O5—Zn2—O3W	89.82 (7)	O4—C10—C9	117.94 (18)
O9^ii^—Zn2—O3W	83.49 (7)	O5—C10—C9	118.18 (17)
O6—Zn2—O3W	173.95 (7)	O6—C11—O7	123.78 (18)
C1—O1—Zn1	144.10 (14)	O6—C11—C12	120.02 (18)
C1—O2—Zn1^i^	119.96 (13)	O7—C11—C12	116.20 (18)
C5—O3—C8	118.82 (16)	C13—C12—C17	118.71 (18)
C10—O5—Zn2	121.42 (13)	C13—C12—C11	119.70 (18)
C11—O6—Zn2	124.43 (15)	C17—C12—C11	121.58 (17)
C11—O7—Zn2^iii^	121.58 (13)	C14—C13—C12	121.23 (19)
C15—O8—C18	117.79 (16)	C14—C13—H13	119.4
C20—O9—Zn2^ii^	131.33 (13)	C12—C13—H13	119.4
C20—O10—Zn1	119.49 (12)	C13—C14—C15	119.43 (18)
Zn1—O1W—H11	112.2 (19)	C13—C14—H14	120.3
Zn1—O1W—H12	109.3 (19)	C15—C14—H14	120.3
H11—O1W—H12	111.0 (16)	O8—C15—C14	124.62 (18)
Zn1—O2W—H21	109 (2)	O8—C15—C16	115.42 (18)
Zn1—O2W—H22	114 (2)	C14—C15—C16	119.96 (19)
H21—O2W—H22	110.4 (16)	C17—C16—C15	120.07 (19)
Zn2—O3W—H31	123 (2)	C17—C16—H16	120.0
Zn2—O3W—H32	108 (2)	C15—C16—H16	120.0
H31—O3W—H32	110.0 (16)	C16—C17—C12	120.56 (18)
O1—C1—O2	122.88 (18)	C16—C17—H17	119.7
O1—C1—C2	118.88 (17)	C12—C17—H17	119.7
O2—C1—C2	118.22 (17)	O8—C18—C19	105.89 (16)
C3—C2—C7	118.70 (18)	O8—C18—H18A	110.6
C3—C2—C1	121.49 (17)	C19—C18—H18A	110.6
C7—C2—C1	119.80 (18)	O8—C18—H18B	110.6
C4—C3—C2	120.90 (18)	C19—C18—H18B	110.6
C4—C3—H3	119.5	H18A—C18—H18B	108.7
C2—C3—H3	119.5	C20—C19—C18	112.35 (16)
C3—C4—C5	120.22 (19)	C20—C19—H19A	109.1
C3—C4—H4	119.9	C18—C19—H19A	109.1
C5—C4—H4	119.9	C20—C19—H19B	109.1
O3—C5—C6	125.13 (18)	C18—C19—H19B	109.1
O3—C5—C4	115.20 (18)	H19A—C19—H19B	107.9
C6—C5—C4	119.68 (18)	O10—C20—O9	122.00 (17)
C5—C6—C7	119.81 (18)	O10—C20—C19	117.89 (17)
C5—C6—H6	120.1	O9—C20—C19	120.09 (17)
			
O10—Zn1—O1—C1	−97.8 (2)	C5—O3—C8—C9	178.69 (18)
O2^i^—Zn1—O1—C1	91.4 (2)	O3—C8—C9—C10	178.60 (17)
O2W—Zn1—O1—C1	−3.0 (2)	Zn2—O5—C10—O4	−2.4 (3)
O1W—Zn1—O1—C1	173.9 (2)	Zn2—O5—C10—C9	178.16 (14)
O7^iii^—Zn2—O5—C10	−9.1 (2)	C8—C9—C10—O4	166.79 (19)
O9^ii^—Zn2—O5—C10	159.36 (15)	C8—C9—C10—O5	−13.7 (3)
O6—Zn2—O5—C10	−109.21 (17)	Zn2—O6—C11—O7	−86.4 (2)
O3W—Zn2—O5—C10	76.81 (17)	Zn2—O6—C11—C12	93.8 (2)
O7^iii^—Zn2—O6—C11	113.26 (16)	Zn2^iii^—O7—C11—O6	−10.7 (3)
O5—Zn2—O6—C11	−120.30 (16)	Zn2^iii^—O7—C11—C12	169.15 (14)
O9^ii^—Zn2—O6—C11	−2.73 (16)	O6—C11—C12—C13	3.2 (3)
O1—Zn1—O10—C20	168.90 (15)	O7—C11—C12—C13	−176.66 (19)
O2^i^—Zn1—O10—C20	−21.89 (19)	O6—C11—C12—C17	−177.8 (2)
O2W—Zn1—O10—C20	78.96 (16)	O7—C11—C12—C17	2.4 (3)
O1W—Zn1—O10—C20	−105.88 (16)	C17—C12—C13—C14	−0.2 (3)
Zn1—O1—C1—O2	−80.4 (3)	C11—C12—C13—C14	178.9 (2)
Zn1—O1—C1—C2	101.1 (2)	C12—C13—C14—C15	1.3 (3)
Zn1^i^—O2—C1—O1	10.1 (3)	C18—O8—C15—C14	0.4 (3)
Zn1^i^—O2—C1—C2	−171.43 (13)	C18—O8—C15—C16	−179.35 (19)
O1—C1—C2—C3	155.8 (2)	C13—C14—C15—O8	179.3 (2)
O2—C1—C2—C3	−22.7 (3)	C13—C14—C15—C16	−0.9 (3)
O1—C1—C2—C7	−24.7 (3)	O8—C15—C16—C17	179.2 (2)
O2—C1—C2—C7	156.8 (2)	C14—C15—C16—C17	−0.6 (4)
C7—C2—C3—C4	0.3 (3)	C15—C16—C17—C12	1.8 (4)
C1—C2—C3—C4	179.79 (19)	C13—C12—C17—C16	−1.4 (3)
C2—C3—C4—C5	−1.3 (3)	C11—C12—C17—C16	179.6 (2)
C8—O3—C5—C6	0.9 (3)	C15—O8—C18—C19	−177.33 (19)
C8—O3—C5—C4	−179.40 (19)	O8—C18—C19—C20	177.22 (17)
C3—C4—C5—O3	−178.8 (2)	Zn1—O10—C20—O9	−6.3 (3)
C3—C4—C5—C6	0.9 (3)	Zn1—O10—C20—C19	172.15 (13)
O3—C5—C6—C7	−179.8 (2)	Zn2^ii^—O9—C20—O10	−169.17 (15)
C4—C5—C6—C7	0.5 (3)	Zn2^ii^—O9—C20—C19	12.4 (3)
C5—C6—C7—C2	−1.5 (3)	C18—C19—C20—O10	25.9 (3)
C3—C2—C7—C6	1.1 (3)	C18—C19—C20—O9	−155.68 (19)
C1—C2—C7—C6	−178.4 (2)		

Symmetry codes: (i) −*x*+1, −*y*+2, −*z*; (ii) −*x*+1, −*y*+1, −*z*+1; (iii) −*x*+1, −*y*+1, −*z*+2.

### Hydrogen-bond geometry (Å, °)

**Table:**

*D*—H···*A*	*D*—H	H···*A*	*D*···*A*	*D*—H···*A*
O1W—H11···O4^iv^	0.83 (1)	1.99 (1)	2.781 (2)	159 (2)
O1W—H12···O4^v^	0.83 (1)	1.94 (1)	2.768 (2)	173 (2)
O2W—H21···O4^vi^	0.83 (1)	2.13 (2)	2.854 (2)	145 (2)
O2W—H22···O7^ii^	0.84 (1)	1.98 (1)	2.776 (2)	158 (2)
O3W—H31···O2^vii^	0.83 (1)	1.91 (2)	2.700 (2)	158 (3)
O3W—H32···O9^viii^	0.84 (1)	2.31 (2)	3.026 (2)	143 (3)

Symmetry codes: (iv) *x*+1, *y*, *z*−1; (v) −*x*+1, −*y*+2, −*z*+1; (vi) *x*, *y*, *z*−1; (ii) −*x*+1, −*y*+1, −*z*+1; (vii) −*x*, −*y*+2, −*z*+1; (viii) *x*−1, *y*, *z*+1.

## References

[bb1] Barbour, L. J. (2001). *J. Supramol. Chem.* **1**, 189–191.

[bb2] Higashi, T. (1995). *ABSCOR* Rigaku Corporation, Tokyo, Japan.

[bb3] Rigaku (1998). *RAPID-AUTO* Rigaku Corporation, Tokyo, Japan.

[bb4] Rigaku (2002). *CrystalClear* Rigaku/MSC Inc., The Woodlands, Texas, USA.

[bb5] Sheldrick, G. M. (2008). *Acta Cryst.* A**64**, 112–122. 10.1107/S0108767307043930 18156677

[bb6] Westrip, S. P. (2010). *J. Appl. Cryst.* **43**, 920–925.

[bb7] Xiao, Y.-H., Gao, S. & Ng, S. W. (2006). *Acta Cryst.* E**62**, m2274–m2276.

